# Cre-Recombinase Dependent Germline Deletion of a Conditional Allele in the Rgs9cre Mouse Line

**DOI:** 10.3389/fncir.2018.00068

**Published:** 2018-09-11

**Authors:** Daniel J. Liput

**Affiliations:** Laboratory for Integrated Neuroscience, National Institute on Alcohol Abuse and Alcoholism, National Institutes of Health, Rockville, MD, United States

**Keywords:** conditional knockout, Cre recombinase, germline, LoxP, RGS9, striatum, medium spiny neuron, basal ganglia

## Abstract

Cre-*Lox*P conditional knockout animals have become a prominent tool to understand gene function in discrete cell-types and neural circuits. However, this technology has significant limitations including off target cre-dependent recombination. The Rgs9cre strain has been used to generate a conditional knockout in striatal medium spiny neurons, but, as presented in the current study, off target recombination in the germline results in nonconditional deletion of *Lox*P alleles. Using a Rem2 conditional allele, germline deletion (GD) was observed in a sex dependent manner. When Cre and *Lox*P alleles were co-inherited from the female parent, 27 of 29 *Lox*P alleles were recombined, but when co-inherited from the male parent, 5 of 36 *Lox*P alleles were recombined. Rem2 expression measured by RT-qPCR confirmed nonconditional recombination in extrastriatal nuclei. Cre-*LoxP* is a powerful technique to modify genomic DNA (gDNA), however careful characterization of these mice is required to confirm control of conditional recombination.

## Introduction

Conditional knockouts using Cre-*LoxP* technology have been instrumental in dissecting the function of proteins in neurophysiology by allowing gene deletion or gene expression with temporal, tissue and cell-type specificity. This technology exploits the function of a bacteriophage P1 topoisomerase, Cre-recombinase (Cre), to recombine genomic DNA (gDNA) that is flanked by a pair of Cre recognition sequences call *Lox*P sites. In many experimental designs, mutant mice containing a floxed (i.e., flanked by *Lox*P) gene of interest are bred with mice expressing Cre driven by a tissue or cell-type specific promoter. These Cre-driver lines are generated by identifying a gene with advantageous expression patterns and inserting the Cre coding sequence into either the endogenous gene locus or a bacterial artificial chromosome (BAC) harboring the gene promoter of interest (the BAC is subsequently introduced into the host genome; Parkitna et al., 2009). Although Cre-*Lox*P conditional knockout mice have been transformative in neuroscience research, significant limitations exist (Schmidt-Supprian and Rajewsky, 2007; Harno et al., 2013). One such limitation is the potential for germline recombination of conditional alleles due to transient Cre expression in the germline (for a detailed description see, Song and Palmiter, 2018), a phenomenon documented with increasing frequency in the literature (Rempe et al., 2006; Zeller et al., 2008; Friedel et al., 2011; Tsai et al., 2012; Kobayashi and Hensch, 2013; Zhang et al., 2013; He et al., 2017; Song and Palmiter, 2018). The current report describes germline recombination in the Rgs9cre line (Dang et al., 2006), which has been widely used to manipulate medium spiny neurons in the striatum.

## Materials and Methods

### Mice

**Figure 1:**
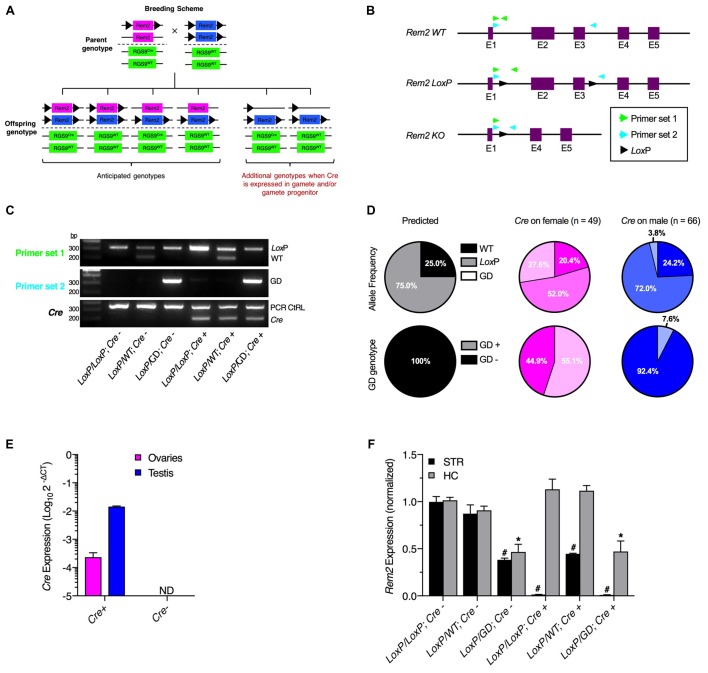
Germline recombination of *Rem2* conditional alleles by Rgs9cre driver mice. **(A)** Summary of anticipated and observed genotypes.** (B)** Schematic of *Rem2* gene showing the wildtype allele, conditional allele with *Lox*P sequences flanking exons two and three, and knockout allele following Cre-recombinase (Cre) dependent deletion. Two primer sets were used to distinguish the three alleles. Primer set one flanked the 5′ *Lox*P site, while primer set two flanked the entire floxed region. Note that the complementary DNA sequence for the reverse primer of Primer set 1 is excised following recombination, which results in failed polymerase chain reaction (PCR) amplification. **(C)** PCR results from genomic DNA (gDNA) purified from ear biopsies. PCR using primer set 1 produced two possible amplicons corresponding to the wildtype (221 bp) and *Lox*P conditional (327 bp) alleles. PCR with primer set 2 resulted in three possible amplicons corresponding to the wildtype (1,915 bp; not shown), *Lox*P conditional (2,065 bp; not shown), and Cre-dependent deleted (338 bp) alleles. The *Cre* PCR reaction contained primers within the Cre coding sequence (200 bp) and PCR control primers (350 bp). **(D)** Allele frequency and germline genotype frequency predicted for the cross, *Rem2*^LoxP/LoxP^*Rgs9*^WT/WT^ × *Rem2*^LoxP/WT^*Rgs9*^Cre/WT^, and observed frequencies when the *Cre* gene was inherited from the female or male parent. **(E)** RT-qPCR from gonad cDNA showing *Cre* expression in *Cre*^+^ mice. **(F)** RT-qPCR from striatum and hippocampus cDNA showing nonconditional Rem2 knockout. ^#^*p* < 0.05 compared to LoxP/LoxP; Cre-STR; *p < 0.05 compared to LoxP/LoxP; Cre-HC. GD, germline deletion. ND, not detected.

All animal studies were conducted in accordance to the National Institutes of Health’s *Guidelines for Animal Care and Use* and all experimental protocols were approved by the National Institute on Alcohol Abuse and Alcoholism Animal Use and Care Committee. Rem2 conditional knockout mice (B6.Cg-*Rem2*^*tm*3551(*T2A-mkate*2)*Arte*^; Liput et al., 2016), which contain *Lox*P sequences flanking exons 2 and 3 (Figure 1B), were bred with Rgs9cre mice, which have a targeted insertion of an IRES-Cre-polyA sequence on the 3′ end of the translated region of exon 19. Mice were bred using the mating scheme, *Rem2*^LoxP,LoxP^*Rgs9*^WT/WT^ × *Rem2*^LoxP/WT^*Rgs9*^Cre/WT^ (Figure 1A), and multiple breeding cages were generated so offspring inherited the *Rgs9*^Cre^ allele from the male or female parent.

### PCR

gDNA was isolated from ear biopsies using the DNeasy kit (Qiagen) and polymerase chain reaction (PCR) was performed using GoTaq DNA polymerase (Promega). For genotyping the *Rem2* alleles, primers were designed to either flank the 5′ *Lox*P site (primer set 1; fwd: 5′-acaaacactcacgcatgcg-3′, rev: 5′-cctacattaggcctagtgagttgg-3′) or the entire floxed DNA sequence containing exons two and three (fwd: 5′-acaaacactcacgcatgcg-3′, rev: 5′-gctacttgctcaactaggagcc-3′, Figure 1B). The genotyping reaction for cre recombinase contained primers for the *Cre* coding sequence (fwd:5′-agcctgttttgcacgttcacc-3′, rev: 5′-ggtttcccgcagaacctgaa-3′) and PCR control primers (fwd: 5′-cctagcacccacccaaagagctg-3′, rev: 5′-ggtcctcactggcagcagctgca-3′).

### RT-qPCR

For *Rem2* mRNA quantification, striata and hippocampi were dissected and placed in Trizol regent for isolation of total RNA using the RNeasy Lipid Tissue kit (Qiagen). For *Cre* mRNA quantification, total RNA was purified from ovaries and testis according to the RNeasy Mini kit (Qiagen). After purification, 500 ng (brain tissues) or 1 μg (ovaries and testis) of RNA was reverse transcribed using the QuantiTect Reverse Transcription kit (Qiagen) and diluted to an estimated 5 ng/μL (cDNA was not diluted for *Cre* reactions). qPCR was performed in triplicate on a StepOnePlus thermocycler using 2 μL (5 μL for *Cre* reactions) cDNA template, Universal Master Mix II, and predesigned TaqMan Gene Expression Assays (all from ThermoFisher Scientific) for *Rem2 (*Mm00600529_m1, spanning exons three and four), *Cre* (Mr00635245_cn), and *actb* (4352933E). Amplification was quantified using the 2^−ΔCT^ method with *actb* serving as the normalization control.

### Statistics

RT-qPCR data was plotted as mean ± SEM and analyzed by two-way ANOVA. For all tests α was set at 0.05.

## Results

To examine germline recombination, male and female mice homozygous for a *Rem2* floxed allele (Liput et al., 2016) were bred with mice heterozygous for both the *Rem2* floxed and *Rgs9cre* alleles (*Rem2*^LoxP/LoxP^*Rgs9*^WT/WT^ × *Rem2*^LoxP/WT^*Rgs9*^Cre/WT^; Figure 1A). Genotypes were determined by PCR using gDNA from ear biopsies. For the *Rem2* allele, two primer sets were designed to flank either the 5′ *Lox*P site (primer set 1) or the entire floxed region (primer set 2; Figure 1B). Using these primer sets and *Cre* primers, PCR product mass revealed six possible genotypes (Figure 1C) as opposed to the four genotypes predicted by Mendelian genetics (Figure 1A). Germline deleted (GD) alleles were detected in many animals by primer set 2, a genotype that cannot be identified by primer set 1 due to excision of the complementary DNA for the reverse primer. Importantly, this result shows that amplifying a single *Lox*P site is insufficient for genotype identification. Allele and genotype frequencies were calculated for offspring generated when the *Rgs9cre* allele was inherited from the female or male parent (Figure 1D). Frequency of the GD allele was 27.6% when *Cre* was inherited from the female parent, while 3.8% when *Cre* was inherited from the male parent. Thus, a GD genotype occurred in 55.1% of offspring generated from a *Cre*^+^ female parent and in 7.6% of offspring generated from a *Cre*^+^ male parent.

If recombined alleles detected in ear biopsies is due to germline recombination, then mRNA should be present in the gonads of Rgs9cre mice. To test this prediction, RT-qPCR was performed using gonadal cDNA from Rgs9cre mice (Figure 1E). *Cre* expression was observed in the gonads of female and male *Cre*^+^, but not *Cre*^−^, mice. Although germline recombination was rarely observed when *Cre* was inherited from the male parent, *Cre* mRNA was more abundant in the male gonads, suggesting that *Cre* is expressed in somatic cells of the testis.

To verify genotypes, RT-qPCR was used to quantify *Rem2* mRNA expression in the striatum, where *Cre* is expressed in the Rgs9cre mouse line, and in the hippocampus, where *Cre* is not expressed in the Rgs9cre mouse line (Figure 1F; Rahman et al., 1999; Dang et al., 2006). Consistent with GD, mice with a *Lox*P/*GD*; *Cre*^−^ genotype had approximately 50% *Rem2* expression in the striatum and hippocampus compared to *Lox*P/*Lox*P; *Cre*^−^ control mice. Additionally, mice with a *Lox*P/*GD*; *Cre*^+^ genotype did not express *Rem2* in the striatum and had approximately 50% *Rem2* expression in the hippocampus.

## Discussion

The current report demonstrates germline recombination of *Rem2* conditional alleles when breeding with the Rgs9cre driver line, which occurred at higher frequency when the *Cre* allele was present on the female parent. As 27 of 29 conditional *Rem2* alleles inherited from the *Cre*^+^ female breeder were GD, breeding in this manner is impractical for generating experimental animals. Although GD occurred with *Cre*^+^ male breeders, the frequency was much lower with 5 of 36 conditional alleles recombined, thus representing a more favorable breeding strategy. A Cre-dependent recombination event resulting in a GD genotype determination could conceivably occur at one of several developmental stages other than in the germline (i.e., in lineage specific stem cells or differentiated somatic cells present in the tissue biopsy). However, none of the 115 mice genotyped for the current study were genotyped GD homozygous, which indicates *Cre* expression and recombination occurred in the germline, prior to fertilization. Additionally, some mice had a GD allele, but were *Cre* negative, further indicating that recombination occurred during gametogenesis prior to generation of the final haploid gametes.

This report, in combination with others, demonstrate the need for careful characterization of Cre-driver lines. In many cases, *Cre* expression patterns are validated by crossing a Cre-driver mouse with a floxed reporter (i.e., LacZ or fluorescent protein) mouse and observing reporter expression in mice from the F1 generation (Madisen et al., 2010). This strategy fails to detect germline recombination because the *Cre* gene and reporter gene are inherited from separate parents, but germline recombination occurs in cases of co-inheritance of the two transgenes from one parent (Schmidt-Supprian and Rajewsky, 2007). In order to screen for germline recombination using a reporter strategy, mice from the F1 generation that have a *Cre*^+/−^; *Reporter*^+/−^ genotype need to be bred with a wildtype mouse and reporter expression examined in the generated offspring (Song and Palmiter, 2018). Although, the utility of this strategy is questionable because Cre may have unequal sensitivity for various floxed conditional alleles (Schmidt-Supprian and Rajewsky, 2007).

As put forth by others (Schmidt-Supprian and Rajewsky, 2007; Kobayashi and Hensch, 2013; Song and Palmiter, 2018), several strategies can be employed to screen for germline recombination. First, genotyping requires the use of primers that flank the entire floxed locus of a conditional gene in combination with primers flanking a single *Lox*P site (Figure 1A for example). This genotyping strategy should be carried out for all conditional alleles due to the possibility of differential sensitivity to Cre-mediated recombination (Schmidt-Supprian and Rajewsky, 2007). Second, male and female breeders harboring the *Cre* gene should be examined to determine the frequency of germline recombination, which can be different between the male and female germline, as reported here for the RGS9cre driver line. Third, PCR from gDNA or RT-qPCR can be performed using an array of tissue types to verify the specificity of recombination (although this strategy alone will inevitably miss recombination in underrepresented cell-types due to non-germline off target Cre activity). Lastly, it is important to report detailed breeding schemes used for generation of experimental animals and methods for validating conditional knockouts so that the possibility of GD deletion can be assessed when interpreting the presented data (Song and Palmiter, 2018).

## Author Contributions

DL designed the experiments, collected and analyzed data and wrote the manuscript.

## Conflict of Interest Statement

The author declares that the research was conducted in the absence of any commercial or financial relationships that could be construed as a potential conflict of interest.
